# Effectiveness of HAPA-based oral hygiene instruction in patients over 65 years of age: a randomized controlled trial

**DOI:** 10.1186/s12903-025-06615-w

**Published:** 2025-07-19

**Authors:** Linn Baumann, Verena Klusmann, Charlène Bamberg, Stefan Rüttermann, Susanne Gerhardt-Szép

**Affiliations:** 1 Department of Operative Dentistry, Carolinum Dental University-Institute gGmbH, J.W. Goethe University, Frankfurt/Main, Germany; 2 Department of Health, Medical and Life Sciences, Health Promotion and Prevention, Furtwangen University, Furtwangen, Germany; 3 Department of Psychology, Psychological Assessment and Health Psychology, University of Konstanz, Konstanz, Germany

**Keywords:** Dentistry, Health behavior theory, Oral health, Patient education

## Abstract

**Objective:**

The aim of the study was to provide oral hygiene instructions tailored to behavioral stages based on the Health Action Process Approach (HAPA) and to assess their impact on oral health compared with standardized instructions in patients aged 65 and older.

**Methods:**

A total of 67 university clinic patients were randomly assigned to an intervention (*n* = 33) and a control group (*n* = 34) and 62 patients, aged 65 to 87 years (*M* = 71.3, *SD* = 5.3), participated in both the baseline and the follow-up visit. To assess oral hygiene, the parameters Gingival Bleeding Index (GBI) and Plaque Control Record (PCR) were recorded at baseline and 12 weeks after instruction. In the intervention group, a questionnaire was used at the beginning of both visits to survey oral hygiene behavior and motivation to change behavior. For both groups, the first visit included a survey on oral hygiene parameters followed by either HAPA-based or standard instruction with demonstration of oral hygiene aids.

**Results:**

Analyses of covariance with baseline control revealed no difference in GBI between the intervention and control group at the 12-week follow-up. Both the intervention group, *t*(29) = 3.91, *p* <.001, *d* = 0.71, as well as the control group, *t*(31) = 3.67, *p* <.001, *d* = 0.65, demonstrated significant improvement in GBI. In contrast, only the intervention group showed a significant improvement in PCR at reevaluation, *t*(29) = 2.29, *p* =.03, *d* = 0.42. Additionally, the intervention group reported a significant increase in motivation and the frequency of interdental care, *t*(29), 6.67, *p* <.001, *d* = 1.22.

**Conclusion:**

This study provides first evidence on the HAPA model being effectively applicable in the context of dental prophylaxis among university clinic patients older than the age of 65. A positive effect on patients’ behavior and oral hygiene could be demonstrated. While general instruction proved effective in reducing gingival bleeding, the theory-based HAPA intervention offered specific advantages in motivation, interdental care, and plaque buildup.

**Trial registration:**

German Study Register of Clinical Studies (DRKS, ID DRKS00030040, German Clinical Trials Register, Date of registration 22.03.2023).

**Supplementary Information:**

The online version contains supplementary material available at 10.1186/s12903-025-06615-w.

## Background

In the European Union (EU), the expected relative share of individuals over the age of 65 will reach an estimated 28.5% in 2050 [1, 2]. With advancing age, underlying clinical conditions become the center of attention in health professions. Oral health, an important factor for quality of life, is often overlooked and only few patients as well as medical professionals are aware of the relationship between oral hygiene and general health [3].

One approach to ensuring high-quality medical care in dentistry is through prevention and health promotion, starting in early childhood—a practice that has been implemented in Germany for over 50 years [4]. As such, advice and education on dental health in combination with demonstrations and exercises on dental care and hygiene are part of routine care during dental visits but are also carried out as part of outreach events in primary education. Multiple studies show that prevention leads to the preservation of the patients’ own teeth and their treatment, even into groups of old age [5, 6]. Long-term tooth preservation in combination with the lengthening of lifespan and advances in living standards however leads to morbidity compression, in which the burden of disease shifts to older ages [7–12]. Accompanying polypharmacy, which—in addition to many other side effects—can in some cases also favor the development of dry mouth (xerostomia) and an altered oral microflora [7, 13, 14]. The reduced saliva flow and the decreasing buffer capacity of saliva lead to an imbalance in the re- and demineralization processes of the toothenamel [15, 16]. In addition, with age, eating patterns and habits often change, as does the sense of appetite and thirst [17]. This can also potentially lead to changes in the composition of saliva and nutrient deficiencies, which are major predisposing factors in the development of caries [18].

As physical and cognitive abilities decline with age, maintaining oral hygiene becomes more complex. Periodontal diseases and the natural aging process contribute to the loss of alveolar bone and gingival tissue, leading to exposure of the cementoenamel junction (CEJ) [19]. This exposure creates a favorable environment for root caries, as plaque tends to accumulate primarily in the proximal areas between two teeth. The exposed root cementum is significantly less mineralized than enamel, making it more susceptible to demineralization making them more vulnerable to acid attacks and lesion formation [20, 21]. Once a lesion develops, it can often progress unnoticed, particularly in the interdental areas, which are challenging to access for both patients and dentists [22]. From a restorative dental perspective, prevention and early management are crucial in this population, as root caries is more prevalent [23, 24]. Given the challenges associated with aging, including reduced dexterity and receding gums, educating older patients on proper interdental cleaning—such as using interdental brushes—is essential for maintaining oral health and preventing root caries [25].

Numerous studies have demonstrated the almost universal applicability of health promotion interventions like Schwarzer’s “Health Action Process Approach” (HAPA) for a number of health behaviors [26, 27]. Widely adopted in health promotion, the HAPA model boasts strong evidence, as consistently demonstrated by meta-analyses [28]. The approach has established planning as a means to bridge the intention-behavior gap, proving particularly effective in situations where intentions fail to translate into action [29]. In a recent conceptual paper, we explained the usefulness of the HAPA model for promoting health aging in particular [30].

Depending on where patients are currently in the process of changing their behavior, a distinction is made between the types of Preintenders, Intenders and Actors [27]. Self-efficacy plays an essential role in Schwarzer’s theory, as it has an extraordinary influence on the formation of an intention and differs depending on willpower and the challenge at hand [31]. Self-efficacy and a positive attitude toward health appear to be proximal predictors of health behaviors [26, 32–38]. As self-efficacy increases, so does the individual’s activity level to indeed change their behavior [34, 37]. The HAPA model distinguishes between a motivational and a volitional phase [39]. In the motivational phase, the person develops the intention to either take a precautionary action or change risky behavior in favor of other behaviors. Self-efficacy and outcome expectations are considered the most important predictors of intentions. Risk expectations also play a large role in motivation, especially among older people [40]. Good intentions, however, do not necessarily guarantee corresponding action. The correlations between intentions and behaviors vary enormously, a phenomenon that has been described as the Intention-Behavior-Gap [41]. While the motivation phase describes what people decide to do, the subsequent action or will phase describes whether they take action and how hard they try and how long they last. Self-efficacy remains an important influencing factor here. Of the strategies that can also be used to increase the level of behavior, planning is the most effective. The special form of recovery self-efficacy ensures that people can resume their behavior even when actions are interrupted or setbacks occur [39].

To date only few studies showed the application of HAPA in dentistry to improve oral hygiene. Those targeted young patients or dentistry students and proved that HAPA-based instruction improved the patients’ oral hygiene [26, 42, 43]. Two studies demonstrated the effect of a planning intervention on dental flossing behavior in mixed aged (18–75 and 18–71 years, respectively) convenient samples [44, 45]. Very recently, a study explored older patients as a primary target group, demonstrating that a HAPA-based intervention increased toothbrushing behavior in an Iranian sample over 60 years old [46]. This study’s scope was limited to comparing different intervention providers, rather than assessing the HAPA’s effectiveness relative to standard care.

Current strategies for enhancing dental hygiene among older adults pinpoint barriers including dentophobia, informational deficiencies, financial constraints, and service accessibility [3]. While these approaches advocate measures to overcome barriers, they fail to address the well-known intention-behavior gap and overlook crucial motivational and volitional processes. Hence, we sought to apply the HAPA-model to dental hygiene instruction specifically for the over-65 age group. We contend that HAPA-based interventions are uniquely positioned to enhance the effectiveness of these oral health instructions. By adeptly overcoming motivational and volitional hurdles, this affordable and straightforward intervention is expected to significantly improve oral health in older patients.

## Methods

### Participants

This randomized controlled trial included patients aged 65 and older as per definition of the EU [2]. A minimum number of 10 remaining interproximal spaces was defined, as the focus of the intervention is on the examination of the interdental areas. To be able to make as true a statement as possible about the oral hygiene of the patients, those who had received professional dental cleaning in the 6 months prior were excluded from the study. A further exclusion criterion was past treatment in the periodontal department to avoid possible bias in interdental brush usage and general knowledge in oral health. Furthermore, patients had to be able and willing to give informed consent and sufficient knowledge of German. No cognitive function tests were performed, and all patients were physically able to visit the university clinic independently and without assistance.

The study was conducted at Carolinum Dental University-Institute gGmbH of J.W. Goethe University (Frankfurt/Main, Germany). Participants were recruited between September and October 2022 and the study was conducted from October 2022 through May 2023.

The study was reviewed by the Ethics Committee of the Goethe University Frankfurt am Main on 08.08.2022 and an ethics vote was granted on 05.09.2022.

### Sample size

The number of cases was planned based Scheerman [42]: A standard deviation (SD) of approximately 25% was expected for the primary outcome measure. A case number of 52 patients is needed to detect a difference of 20% at a significance level of 5% with a statistical power of 80% using a two-sided 2-sample t-test. Considering the follow-up time of three months and a drop-out rate of 20%, the required minimum case number was 64 participants.

### Randomization and blinding

This study was designed as a randomized controlled trial with a parallel-group structure, in which participants were randomly assigned in a 1:1 ratio to one of two instructional groups.

The random allocation sequence was generated using simple randomization in “R” statistical software by a researcher who was neither involved in patient instruction nor outcome assessment. The group assignments were prepared in advance and placed in named, sealed envelopes along with all the additional material and stored securely. These envelopes were opened by baseline investigators immediately prior to the instructional session, in order to deliver the appropriate intervention. Consequently, baseline instructors could not be blinded. As the instructional content differed between groups and included the completion of the HAPA questionnaire for the intervention group, participants could also not be blinded to group allocation. This is also due to the necessity to disclose details about the trial while obtaining consent for the participation as required by the ethics commission. Secondary clinical investigators evaluating the oral hygiene indices at follow-up remained blinded to group allocation to minimize biased reports. The secondary clinical investigators evaluating the oral hygiene indices at the follow-up appointment remained blinded to group allocation to minimize biased reports.

### Statistical analysis

Baseline characteristics of the intervention and control groups were compared using unidimensional analysis of variance (ANOVA). Multivariate analysis of variance (MANOVA) was employed to compare baseline scores across the three HAPA stages for multiple dependent variables: GBI, PCR, frequency of brushing, and interdental brush use.

To evaluate the impact of our intervention on outcome measures over time, we conducted analyses of covariance (ANCOVA) on 12-week follow-up scores, controlling for baseline and age. Chi^2^-test was performed to compare stage groups and examine the frequency of participants in the HAPA stage groups. Repeated measures analysis of variance (ANOVA) was conducted to assess changes in HAPA measures in the intervention group from baseline to follow-up.

Follow-up scores across the three HAPA stages within the HAPA group, while controlling for baseline scores, were analyzed using multivariate analysis of covariance (MANCOVA) with stage (Preintender vs. Intender vs. Actor) as a between-subjects factor and GBI, PCR, frequency of brushing, and interdental brush use as dependent variables.

Significance levels were conventionally fixed at *p* <.05, and all analyses were conducted using SPSS (Version 30). Bonferroni correction was applied to control for possible alpha error cumulation due to multiple testing and is specified in detail for all analyses in which several dependent variables are considered simultaneously (i.e., dividing *p* <.05 by the number of multiple tests conducted). Effect sizes (Eta-squared η^2^ for analyses of variance, Cohen’s *d* for t-tests) were designated to provide a comprehensive understanding of observed effects.

No interim analyses were planned or conducted, and no formal stopping guidelines were established due to the low risk associated with the intervention and the trial’s brief duration.

### Design / Measures

The study distinguishes an intervention group receiving the HAPA-based instruction and a control group receiving a standardized oral hygiene instruction.

Both groups differed only in their specific instructional content and were treated identically otherwise. Patients played no role in defining research questions or outcome measures and were not involved in developing plans for recruitment, design, or implementation of the study. This study adheres to CONSORT 2025 guidelines.

### Clinical assessment of oral hygiene

For this study, Plaque Control Record (PCR) was used to assess plaque formation. It involves staining of the microbial plaque with a plaque detector to visualize plaque on the tooth surface [47]. Six surfaces (vestibular and oral, each distal, central and mesial) were assessed per tooth and the percentage was then calculated from the sum of the plaque-positive areas in relation to the total number of areas assessed.

As a long-term indicator the Gingival Bleeding Index (GBI) was recorded. For this index, each sulcus is probed, and the presence of bleeding is determined after 10 s. As with the PCR index, the GBI involves the assessment of six surfaces per tooth. For each surface, it is indicated whether it is bleeding or not, regardless of the intensity. The value was calculated analogously to PCR [48].

### Intervention

Sessions lasted approximately 65 min. The initial step involved examining the oral mucosa and dental hard tissue, along with identifying dental, oral, and maxillofacial diseases (e.g., carious lesions, insufficient dentures). Then, GBI and PCR values were recorded by previously calibrated clinical investigators. To calibrate GBI measurements, investigators participated in multiple training workshops. These workshops focused on maintaining a consistent probing pressure of 20–25 g using a calibrated scale. For calibration of the PCR assessment, test patients were used.

After that two distinct intervention approaches were applied. The control group received a standardized oral health instruction and the intervention group received additional HAPA-based instructions. Following the instruction, all patients received a professional teeth cleaning.

Clinical examinations and all patient instructions were performed by trained and calibrated dentists.

#### Standard protocol: control group

Participants of the control group were given a standard oral hygiene instruction after the recording of the oral health indicators. This included demonstrations on a dental care model and in the oral cavity using oral hygiene aids to improve the effectiveness of the patient’s home oral hygiene efforts. The focus was on the demonstration and correct application of interdental hygiene aids, such as dental floss and above all individual intraoral adjustment of interdental brushes. At the end of the session, the investigators gave patients the adjusted interdental brushes along with a record of the determined ISO sizes.

#### Intervention group

Patients of the intervention group received a questionnaire before the oral examination and were given additional 15 min to answer it in the treatment room. The questionnaire was based on a validated one on dental flossing introduced by Schwarzer et al. [26]. For this study, the use of dental floss was replaced by the use of interdental brushes. It assessed the patient’s oral hygiene behavior as well as motivation and attitude towards the use of interdental brushes at the present and in the future.

A total score of 0–69 could be achieved. Different scores were assigned to HAPA’s three behavioral types: 0–22 points were classified as Preintenders, 23–46 points as Intenders and 47–69 points as Actors.

The following session was identical to the control groups protocol, including the clinical assessment. The instruction had the same content, but different emphases, which were weighted depending on the activity and motivation of the patient as determined by the questionnaire. Based on the classification of the behavioral stage into Preintender, Intender and Actor, the patients received tailored instructions. For all three stages, the instruction ended after 15 min with the patient writing a personal goal, which was given to them as a reminder along with the adjusted interdental brushes and ISO size record.

The focus of the Preintender instruction was to explain the basics of oral hygiene and to deepen the knowledge of prophylaxis. The instructional focus was on teaching patients how to use oral hygiene aids correctly. This included demonstrating the proper use of toothbrushes, whether manual or electric. Patients were shown toothbrushing techniques using a dental care model and the importance of interdental cleaning, specifically the correct use of interdental brushes, was emphasized. This instruction also provided the multifactorial explanation of caries etiology and risk education: “How does caries develop and how can it be counteracted?” and “What are the consequences of caries?” explaining the local and systemic effects.

For the Intender stage, the focus of the instruction was also on theoretical cleaning basics. Identified knowledge gaps were addressed by providing the necessary supplementary information. In addition, the focus here was on the concrete planning for behavioral change. The instruction session was used to aid the patient to improve their brushing behavior and to make them aware of their own behavior. Patients were encouraged to reflect on and reconsider their current behavior, identifying strategies for modifying it, and set realistic, short-term goals.

If the patient was assigned to the Actor stage, the focus was on implementation assistance, planning, and on behavior maintenance. The patients already had a strong base knowledge, so the instruction for this stage did not go into detail about the etiology and pathogenesis of caries and the explanation of oral hygiene strategies. The instruction addressed the questions such as, “What are my short- and long-term behavior change goals?”, “What obstacles might occur during my behavior change?”, and “How can I counteract the obstacles to achieve my goal?”.

### Follow-up

Primary and secondary outcomes were evaluated 12 weeks after the first appointment at 12-week follow-up visit. Except for handing out the questionnaire in the intervention group prior to the evaluation, the follow-up visit was conducted in the same way in both groups and took about 30 min. At the appointment, the GBI and PCR indices were recorded again. During the second appointment, however, the parameters were collected by a different, blinded to study conditions and previously calibrated clinical investigator.

The original investigators then discussed the oral hygiene results with the patient and individual remotivation took place. For remotivation, all patients were again given individual instructions to resume or enhance their oral care habits. Specific instruction based on their individual GBI and PCR values were provided, the importance of careful oral hygiene was reemphasized and individual barriers to proper care were identified and addressed. In the intervention group, additional reference was made to the goals previously set in writing.

### Harms

The interventions consisted of standard professional teeth cleaning, assessment of oral hygiene indices and delivery of oral health instructions via two distinct approaches. Both professional teeth cleaning and oral hygiene assessment are routine, non-invasive and low risk procedures in dental practice. At the follow-up visit, participants were asked about any unexpected effects. No adverse events were reported.

## Results

**Fig. 1:**
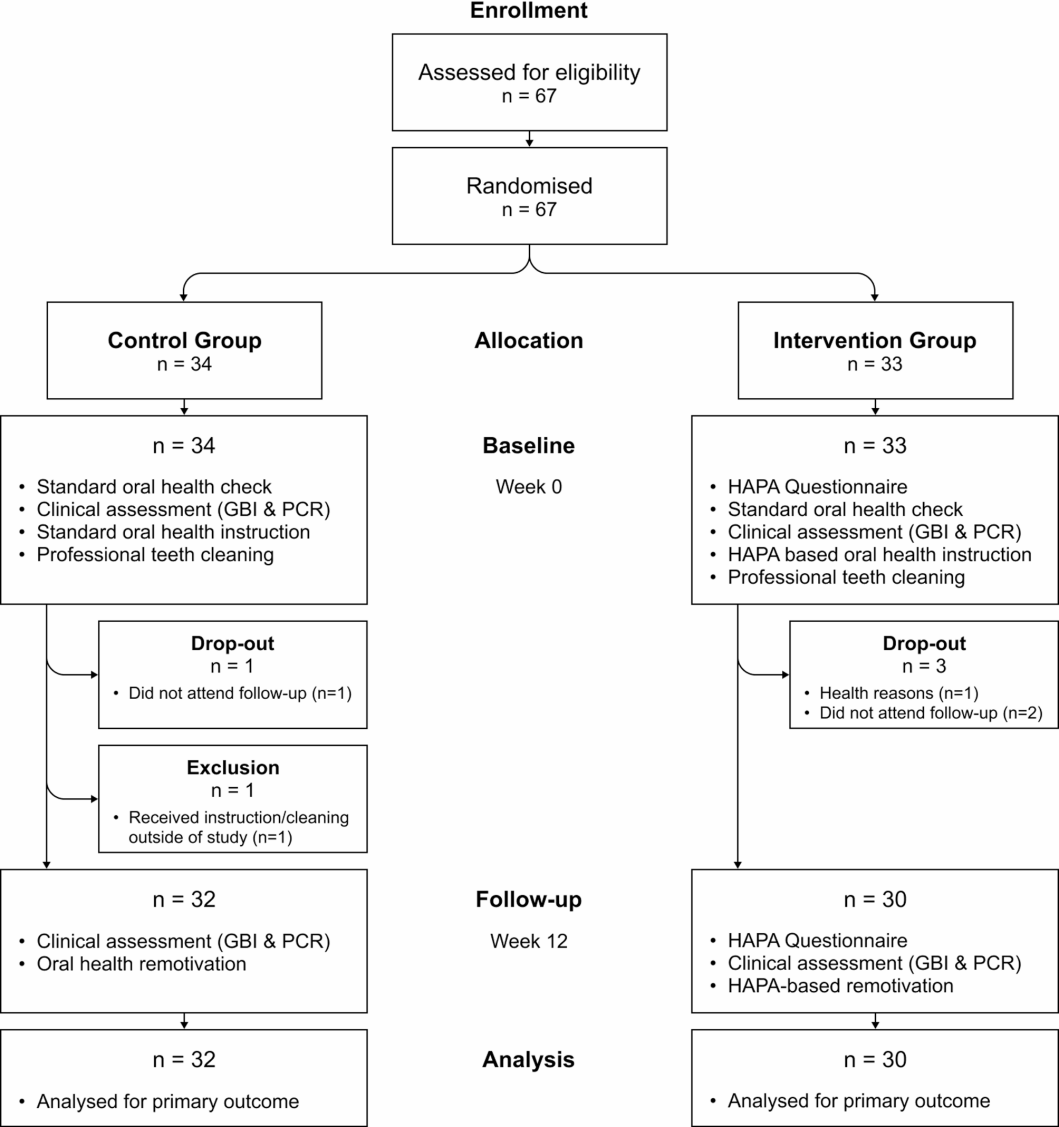
Study procedures

A total of 67 participants were recruited of which 62 showed up for the second appointment (Fig. 1). The drop-out rate was 7.5%. The loss of five patients had the following reasons: three could not be reached by phone or mail to arrange the second appointment, one patient dropped out due to health reasons and one patient was excluded because they received oral hygiene instruction between baseline and follow-up outside of the study.

All eligible patients in both groups received group-specific instructions, as previously described, and did not receive any concomitant oral care. No adverse events or unintended outcomes were observed or reported. The mean time between both appointments was 12.29 weeks (SD 1.21). The earliest reevaluation occurred after 10 weeks and the latest after 19 weeks. A total of 29 male and 33 female patients participated in the study. The mean age was 71.3 for male and 71.2 years for female patients.

**Table 1:** Unidimensional analysis of variance (ANOVA) on baseline scores comparing intervention vs. control group

	Total (*n* = 62)	Intervention group (*n* = 30)	Control group (*n* = 32)	F-Test results (ANOVA)
	Mean ± SD	Mean ± SD	Mean ± SD	
Age	71.3 ± 5.3	70.53 ± 4.29	71.94 ± 6.09	*F*(1,60) = 1.09, *p* =.30
Interprox. spaces	22.5 ± 4.9	22.5 ± 4.55	22.47 ± 5.28	*F*(1,60) < 0.01, *p* =.98
GBI in %	10.45 ± 8.09	9.5 ± 5.76	11.34 ± 9.80	*F*(1,60) = 0.80, *p* =.37
PCR in %	60.82 ± 17.49	62.27 ± 17.05	59.47 ± 18.06	*F*(1,60) = 0.39, *p* =.53

Note. *SD* = standard deviation. Variance homogeneity concerning all variables was confirmed via Levene-test (all *p* >.05)

The mean interproximal space count was 22.5 (SD 4.9) across both groups, with minimal variation between them (see Table 1). Baseline levels of GBI and PCR were comparable in both groups.

**Table 2:** Baseline scores comparing preintender, intender and actors via multivariate analysis of variance (MANOVA)

	Preintender (*n* = 3)	Intender (*n* = 14)	Actor (*n* = 13)	F-Test results (ANOVA)
	Mean ± SD	Mean ± SD	Mean ± SD	
Age	70.0 ± 4.36	71.79 ± 5.29	69.31 ± 2.72	*F*(2,27) = 1.16, *p* =.33
Interprox. spaces	20.67 ± 6.35	20.86 ± 4.94	24.69 ± 2.75	*F*(2,27) = 3.05, *p* =.064
GBI in %	13.0 ± 1.0	8.79 ± 3.64	9.46 ± 7.87	*F*(2,27) = 0.65, *p* =.53
PCR in %	55.33 ± 21.73	64.64 ± 15.83	61.31 ± 18.23	*F*(2,27) = 0.39, *p* =.68
Frequency of brushing	10.33 ± 3.51	11.71 ± 3.91	14.54 ± 3.46	*F*(2,27) = 2.73, *p* =.084
Use of interdental brushes	0 ± 0	2.93 ± 3.05	6.77 ± 5.09	***F*(2**,**27) = 5.04**, ***p*** =.014*

Note. *SD* = standard deviation. *indicates a significant difference across stages (higher use of interdental brushes in actors) at *p* <.05

Comparing baseline scores between groups across all three HAPA stages (see Table 2) reveals that those in the Actor stage showed a significantly higher use of interdental brushes with an average use of *M* = 6.77, *SD* = 5.09 times weekly, *F*(2,27) = 5.04, *p* =.014. This is logically appropriate to stage algorithm.

**Table 3:** Analysis of covariance (ANCOVA) on GBI, PCR, follow-up scores comparing intervention and control group

ANCOVA main effect	Group	M (SD)		Change ΔM (SD)	Within-group contrast (t-tests) Baseline to follow-up			
		Baseline	Follow-up		t	df	*p*	d
GBI F(1,57) = 0.15, *p* =.70, η2 = 0.003	Intervention group	9.5 ± 5.76	5.70 ± 4.13	–3.80 ± 5.32**	3.91	29	< 0.001	0.71
	Control group	11.34 ± 9.80	5.56 ± 3.93	–5.78 ± 8.91**	3.67	31	< 0.001	0.65
**PCR** **F(1**,**57) = 5.19**, *p* =.026,** η2 = 0.083**	Intervention group	62.27 ± 17.05	57.00 ± 15.63	–5.27 ± 12.62*	2.29	29	0.030	0.42
	Control group	59.47 ± 18.06	60.69 ± 15.33	1.22 ± 14.55, n.s.	–0.47	31	0.319	–0.08

Note. *M* = mean, *SD* = standard deviation. Variance homogeneity concerning all variables was confirmed via Levene-test (all *p* >.05). **indicates a significant change over time at *p* <.001; *indicates a significant change over time at *p* <.01

The intervention and control group did not differ in terms of GBI, as indicated by a non-significant ANCOVA main effect, *F*(1,57) = 0.15, *p* =.70, η^2^ = 0.003 (no significant between group difference). Both groups showed a significant reduction of GBI scores at follow-up, qualified by a significant within-group contrast with moderate sizes of effect (Cohen’s *d* > 0.5), *t*(29) = 3.91, *p* <.001, *d* = 0.71, for the intervention group and *t*(31) = 3.67, *p* <.001, *d* = 0.65, for the control group (cf. Table 3). As for PCR, the intervention group scored significantly lower than the control group as qualified by a significant ANCOVA main effect, *F*(1,57) = 5.19, *p* =.026, η^2^ = 0.083 (significant between-group difference). Only the intervention group had a significantly lowered PCR score at follow-up, as indicated by a significant within-group contrast, *t*(29) = 2.29, *p* =.03.

**Table 4:** Stage group descriptives and participant frequencies pre vs. post intervention (chi^2^-test)

HAPA stages at baseline	Preintender (*n* = 3)	Intender (*n* = 14)	Actor (*n* = 13)
Frequency of brushing per week *M (SD)*	10.53 ± 3.51	11.71 ± 3.91	14.54 ± 3.46
Use of interdental brushes per week *M (SD)*	0	2.93 ± 3.05	6.77 ± 5.09
Interprox. Spaces *M (SD)*	20.67 ± 6.35	20.86 ± 4.94	24.69 ± 2.75
Age *M (SD)*	70.00 ± 4.36	71.79 ± 5.29	69.31 ± 2.72
Gender *n* = 17 women (56.7%) χ^2^(2) = 5.81, *p* =.06	3 (23% of men) 0 (0% of women)	5 (38.5% of men) 9 (52.9% of women)	5 (38.5% of men) 8 (47.1% of women)
HAPA stages at posttest (n, % of change from baseline), χ^2^(2) = 7.10, *p* =.019	0 (–100%)	10 (–50% of baseline n)	20 (+ 53.8% compared to baseline)

Note. *M* = mean, *SD* = standard deviation. *indicates a significant change over time at *p* <.01

Investigation of characteristics of the patients between the groups across the three HAPA stages showed that there were some more women in the higher stages of motivation (Intender) and volition (Actor), but cell numbers were low given the small sample size and there were no significant differences in the gender distribution (see Table 4).

Change of participants in stage groups from baseline to follow-up (post intervention) was significant, χ^2^(2) = 7.10, *p* =.019. The three Preintenders progressed to Intenders and seven of the 14 patients who had been Intenders at baseline, turned into Actors at follow-up. As a result, the number of Actors significantly increased from *n* = 13 at baseline to *n* = 20 at follow-up, representing a 53.8% increase. The absolute decrease in Intenders was 50%.

**Table 5:** Repeated measures ANOVA baseline to follow-up in intervention group

Measure (*n* = 30)	M (SD)		Change ΔM (SD)	Wilks-Lambda F-test on baseline to follow-up			
	Baseline	Follow-up		F	df	*p*	η^2^
Frequency of brushing per week	12.8 ± 3.9	13.1 ± 4.3	0.827 ± 2.05	0.51	29	0.482	0.02
Use of interdental brushes per week	4.30 ± 4.52	9.93 ± 4.79	5.63 ± 4.63**	44.46	29	< 0.001	**0.61**
Intention	2.82 ± 0.94	2.98 ± 0.62	0.17 ± 0.94	0.94	29	0.340	0.03
Outcome expectancy	2.67 ± 0.80	3.14 ± 0.68	0.48 ± 0.64**	16.96	29	< 0.001	0.37
Risk perception	2.97 ± 0.88	3.24 ± 0.97	0.28 ± 1.08	2.00	29	0.168	0.06
Action self-efficacy	2.73 ± 0.89	3.51 ± 0.52	0.78 ± 0.92**	21.67	29	< 0.001	**0.43**
Coping self-efficacy	2.97 ± 0.69	3.41 ± 0.57	0.44 ± 0.72*	11.49	29	0.002	0.28
Action planning	2.81 ± 0.87	3.34 ± 0.50	0.53 ± 0.79**	13.82	29	< 0.001	0.32
Coping planning	2.51 ± 0.91	2.85 ± 0.68	0.34 ± 0.78*	5.92	29	0.021	0.17
Behavioral control	2.51 ± 0.92	3.57 ± 0.47	1.06 ± 0.91**	40.74	29	< 0.001	**0.58**

Note. *M* = mean, *SD* = standard deviation, df = degrees of freedom, η2 = Eta-suqared effect size with values above 0.14 commonly regarded as large effects. **indicates a significant change over time at *p* <.001; *indicates a significant change over time at *p* <.01. Largest effect sizes printed in bold. Bonferroni correction was applied to control for possible alpha error cumulation due to multiple F-tests: defining a significance level of 5% this was devided by the no. of *F*-test (10), which means 0.05/10 = 0.005; at least one effect should be significant at 0.5%, which is true for the data; hence results of repeated-measures ANOVA can be regarded as solid

Results of repeated measures ANOVA on baseline to follow-up regarding motivational and behavioral reports in the intervention group show significant increases in nearly all HAPA measures, except for intention formation and risk perception (see Table 5). The largest effect appears in the primary outcome, that is, self-reported use of interdental brushes per week, *F*(1,29) = 44.46, *p* <.001, η^2^ = 0.61, followed by behavioral control, *F*(1, 29) = 40.74, *p* <.001, η^2^ = 0.58, and action self-efficacy, *F*(1,29) = 21.67, *p* <.001, η^2^ = 0.43. The latter two are important predictors of health behavior initiation and maintenance of a newly established behavior.

**Table 6:** Multivariate analysis of covariance (MANCOVA) on follow-up scores comparing HAPA-stages, controlling for baseline scores

	Preintender (*n* = 3)	Intender (*n* = 14)	Actor (*n* = 13)	F-Test results (ANOVA)
	Mean ± SD	Mean ± SD	Mean ± SD	
GBI in %	2.67 ± 1.16	5.93 ± 4.37	6.15 ± 4.20	*F*(2,23) = 1.96, *p* =.16
PCR in %	56.0 ± 6.08	60.43 ± 15.25	53.54 ± 17.47	*F*(2,23) = 1.26, *p* =.30
Frequency of brushing	9.67 ± 3.79	11.50 ± 3.35	15.54 ± 4.10	*F*(2,23) = 1.71, *p* =.20
Use of interdental brushes	9.33 ± 4.04	7.36 ± 3.30	12.85 ± 4.90	*F*(2,23) = 3.31, *p* =.055^†^

Note. *SD* = standard deviation. Variance homogeneity concerning all variables was confirmed via Levene-test (all *p* >.05). † indicates a tendency at a level of less than 6% probability of error, although not reaching statistical significance, *p* =.055

A MANCOVA on follow-up scores across HAPA stages; controlling for baseline scores, revealed no significant differences between the groups in GBI, PCR, and frequency of brushing (all *p* >.16). A tendency was found in the use of interdental brushes, with Actors reporting higher usage than Preintenders at follow-up, *F*(2,23) = 3.31, *p* =.055 (see Table 6).

## Discussion

To date, the Health Action Process Approach (HAPA) model has been applied to various health behaviors like physical activity, medication adherence and general dental visits in older adults [49]. However, its potential in promoting oral health behavior in this population remains largely unexplored. Previous studies have predominantly focused on younger populations and specific oral care aspects such as flossing [42, 44]. This randomized controlled study demonstrates the applicability and effectiveness of a HAPA-based intervention in older patients, with a significant increase in interdental brush usage and improved clinical measures of oral hygiene.

Both control and intervention groups achieved a significant improvement in the GBI value at follow-up of medium effect size. These results indicate that both forms of instruction have a positive influence on the patients’ gingival health, regardless of their specific specifications. The observed improvements in gingival health suggest that even standard instructions can promote beneficial outcomes. This result is consistent with the study of Stenman et al. [50] where the intervention group did not achieve significant differences in the improvement of the bleeding index compared to the control group receiving standard instruction.

The reduction of PCR was however significantly higher in the intervention group compared to the control group. At follow-up, the intervention group had a lower PCR score (*M* = 57, *SD* = 15.63) compared to the control group (*M* = 60.69, *SD* = 15.33), with no significant improvements in the PCR score in controls. The significant reduction in the PCR value may be due to the HAPA instruction, which resulted in an improvement in oral hygiene. It is important to note, that PCR provides a snapshot of oral hygiene, as it is a dichotomous index. In the context of clinical relevance, a change of 5% in PCR can indicate a significant individual improvement in a patient with an already low PCR, as improvement is harder when the baseline PCR is low.

Beyond clinical measures, the intervention produced significant behavioral changes. A significant increase of the reported use of interdental brushes per week (Δ*M* = 5.63, *SD* = 4.63) was observed, with a large effect size, in the intervention group. While patients at the first appointment reported interdental brush use with an average of 4.3 times per week, at the second appointment participants reported a frequency of 9.9 times per week on average. This is the primary behavioral outcome and reflects a significant enhancement in patients’ oral hygiene practices. The observed improvements in interdental cleaning behavior and the clinical outcomes are strongly linked to the principles of the HAPA model. The intervention directly addressed key components of motivational and volitional phases, providing tailored feedback, interdental brushes and written objectives aimed to enhance participants’ self-efficacy beliefs regarding their ability to perform interdental cleaning effectively [51, 52]. These findings align with previous research highlighting the relationship between individual self-efficacy and toothbrushing frequency aligns with the results of previous studies [42, 53, 54]. However, as the usage of interdental brushes was self-reported, it is possible that there was recall bias. Compared to the 6th German Oral Health Study (DMS 6), a population representative socioepidemologic monitoring of oral health care status in Germany, their baseline characteristics for age group 65–74 year olds, showed a similar reporting to that of our baseline. A total of 83,4% of 797 participants reported a brushing frequency of ≥ 2 times daily. Interdental cleaning was reported at 61.9% less than once and 38,1% at more than once daily. The data of our intervention group therefore does not appear to considerably deviate from the DMS 6, at least on average [24].

Detailed analyses of our follow-up questionnaires of the intervention group revealed notable improvements across all measured motivational and behavioral variables except for risk perception and intention, indicating enhanced dental health behaviors and social-cognitions among the participants. Substantial, strongly significant increases in action self-efficacy (Δ*M* = 0.78) and behavioral control (Δ*M* = 1.06) were observed, both of which are crucial for initiating and maintaining health behaviors [39]. The HAPA model proposes that intentions to engage in a particular behavior are preceded by risk perception, outcome expectancies, and self-efficacy beliefs, while the translation of these intentions into action is facilitated by action and coping planning [39]. Our findings support this framework, demonstrating the model’s applicability in promoting oral health behaviors. The weak change of risk perception (Δ*M* = 0.28) between baseline and follow-up could be explained by an already heightened awareness that could be due to the fact that the patients were recruited at a university dental clinic, suggesting that participants may have had pre-existing knowledge of oral health risks.

The HAPA based instruction effectively helped participants progress through the HAPA stages, with a substantial number of patients moving towards the action stage at follow-up. At baseline, three participants were Preintenders, 14 were Intenders, and 13 were Actors. Following the intervention, none remained in the Preintender stage, indicating that a reasonable amount of participants had made substantial progress towards intention formation or action initiation. Notably, among the initial Intenders (*n* = 14), seven transitioned to the Actor stage at follow-up, reflecting a 50% reduction in the number of intenders and a significant increase in Actors from *n* = 13 at baseline to *n* = 20 post-intervention, which means an absolute increase of 53.8%. This shift suggests that the intervention effectively facilitated the participants’ progression through the motivational phase (preintention to intention) and into the volitional stage (intention to action), ultimately enhancing their commitment to adopting and maintaining improved dental hygiene practices. The emphasis on goal-setting and action planning within the intervention likely facilitated this progression, providing participants with the necessary tools and support to overcome barriers to behavior change [51, 52]. While the relatively low number of preintenders at baseline might be explained by the assumed higher motivational levels in a sample recruited from a university dental clinic, the fact that they had to actively agree to participate in the study might also contribute to a heightened interest in improving oral health.

The comparison of the follow-up scores within the intervention group and across the three HAPA stages while controlling for baseline scores shows no significant differences in GBI, PCR and frequency of brushing, but there was a tendency of higher use of interdental brushes among Actors at follow-up. This could suggest that participants who had progressed to the Actor stage were more likely to adopt and maintain this critical oral hygiene practice. In a study by Jönsson et al. personal goals of the intervention group were documented in a written commitment after the instruction and the majority of patients in their study’s intervention group reported that this written commitment positively influenced their oral care habits at follow-up [55]. While we also implemented written commitments in our study, we did not assess their impact on patient behavior post-intervention. Nevertheless, Jönsson et al.’s findings suggest that such commitments could have contributed to the increased interdental brush usage observed in our intervention group. The fact that no significant change in toothbrushing frequency was reported is in line with the study by Kakudate et al., in which the frequency of toothbrushing surveyed did not increase significantly at the time of re-presentation either [54]. The reason for this is seen in the already high frequency of toothbrushing at the beginning of the study.

The age range of the participating patients (65–87 years) aligns with a large portion of the older adult population. The study therefore provides insight into the applicability of the HAPA-model across this diverse age group that—despite the shared designation of “older adult”—reflect a cross-section of different life stages. The similar gender and age distributions across both study groups suggest that the findings may be applicable to populations with comparable demographic characteristics.

Across all participants the mean number of proximal spaces was 22.5, far exceeding the required minimum of 11 proximal spaces defined as inclusion criteria of this study. The mean value of > 22 interproximal spaces even exceeds the published figures of the sixth German oral health study, where younger old-adults (65–74 years) have an average of 18.8 teeth, which translates to 18 or fewer proximal spaces [24]. However, this could suggest that the patients in our study were particularly oral hygiene-conscious. A possible explanation for this observation would be an assumed increased dental health awareness in patients at a university dental clinic.

Our re-evaluation period was defined as approximately 12 weeks. Most existing dental studies aimed at behavioral change have defined an interval of three months for re-evaluation [42, 56–58]. The basis for the definition of this period was the assumption that the behavioral change and thus the implementation of new habits in everyday life must be carried out over a certain period of time in order to be applied routinely and automatically [59]. Three months were described as sufficient for changes in oral hygiene in a study by Axelsson and Lindhe [60]. However depending on the action and the individual attitude and discipline, this time interval can also vary [61]. As behavior changes in any field can fade without continued support [62], it would be reasonable to examine the participants again at a later point in time to assess the long-term sustainability of the observed behavioral changes. One of the aims of the study was to simplify the model and make it part of a routine check-up so that remotivation could be implemented during regular dentist visits every 6 to 12 months.

This study does have limitations that should be considered. The sample size of 64 was relatively modest, which may limit the generalizability of the findings. This is particularly relevant in the analysis of effects across HAPA stages, as statistical power was reduced due to small subgroup sample sizes. A larger, more diverse sample, potentially including patients with lower initial motivation levels could provide a more comprehensive understanding of the observed effects. However, for the context of a university hospital, the participant structure is realistic and argues for high ecological validity. Furthermore, the study duration was limited to 12 weeks, and longer-term follow-up is needed to assess the sustainability of the observed behavioral changes. In future studies, post-intervention assessments should be conducted. All information in the questionnaire relied on self-reported information, which may introduce recall bias. In future studies, the control group should also report on the use of interdental brushes and the frequency of brushing. Additionally, objective measures, such as electronic brushes with usage tracking capabilities, could be incorporated to mitigate bias. For the evaluation of GBI, constant-force probing devices could be used to further standardize measurements. Participants were unable to be blinded because disclosure of intervention details was required for informed consent, which could have introduced expectation-bias. We did not assess socioeconomic and cognitive status in this study, which is however known to influence oral hygiene behaviors and outcomes [63, 64]. All participants were recruited at a university dental clinic, which limits the generalizability of these findings. Future research could address these aspects to provide a more complete picture. Given that the evaluation of oral health status is inherently subjective and can be affected by inter- and intra-examiner variability, we minimized these effects by keeping the number of practitioners as small as possible. All selected practitioners were calibrated as described previously.

## Conclusions

This study demonstrates the efficacy of a HAPA-based intervention to effectively promote interdental cleaning and improve oral health outcomes among older adults.

By addressing both motivational and volitional factors, our intervention successfully translated intention into action, leading to significant improvements in self-efficacy, interdental cleaning frequency, and clinical measures of oral health. These findings contribute to the growing evidence supporting the applicability of the HAPA model across diverse health behaviors and age groups. They also highlight the opportunity of empowering older adults to trust in their ability to self-manage their oral health, thereby offering a promising approach to reducing the burden of oral disease in this vulnerable population.

In practice, dental professionals can incorporate HAPA principles into their counseling and education as a potentially cost-effective strategy to support positive behavioral change and improve both oral health and quality of life for older patients. However, this study provides insight into a small sample over a relatively short timeframe, so further research is needed with larger, more diverse and representative samples, extended timeframes, and more comprehensive objective behavioral assessments. Our results provide an important first step and proof of principle for developing effective interventions tailored to the specific oral health-care needs of older adults. Additionally, further research should focus on developing and evaluating simplified guidelines for incorporating HAPA based instruction in routine practice for older patients.

## Electronic supplementary material

Below is the link to the electronic supplementary material.

Supplementary Material 1

## Data Availability

All data generated and analyzed during this study are included in this published article and its supplementary files.

## Abbreviations

HAPA: Health Action Process Approach
GBI: Gingival Bleeding Index
PCR: Plaque Control Record
EU: European Union
CEJ: Cementoenamel Junction
SD: Standard Deviation
ANOVA: Analysis of Variance
MANOVA: Multivariate Analysis of Variance
ANCOVA: Analysis of Covariance
MANCOVA: Multivariate Analysis of Covariance
